# Meeting the needs of older adults living in long‐term care homes through co‐design of a Virtual Care Planning ToolKit

**DOI:** 10.1002/alz70858_102270

**Published:** 2025-12-26

**Authors:** Marie‐Lee Yous, Denise M Connelly, Nancy Snobelen, Lillian Hung, Melissa Babcock, Debra Lernout‐Banks, Joanne Collins, Maureen O’Connell, Cathy Sturdy‐Smith, Deirdre Finnigan, Cassidi Nother

**Affiliations:** ^1^ McMaster University, Hamilton, ON, Canada; ^2^ The University of Western Ontario, London, ON, Canada; ^3^ WeRPN (Registered Practical Nurses Association of Ontario), Mississauga, ON, Canada; ^4^ University of British Columbia, Vancouver, BC, Canada; ^5^ Vision 74 inc., Sarnia, ON, Canada; ^6^ Copper Terrace, Chatham, ON, Canada; ^7^ Pieces Canada, Windsor Junction, NS, Canada; ^8^ Queen's University, Kingston, ON, Canada

## Abstract

**Background:**

Health/safety restrictions to control COVID‐19 in long‐term care (LTC) homes were very challenging for residents, their families, administrators, and staff. Mental and physical well‐being of residents declined with the imposed social isolation, workforce shortages, and loss of informal care provision by family/care partners. Unmet needs of older adults living with dementia manifest as behavioural expressions (e.g., irritable, agitated or aggressive behaviour). The [PIECES approach] (**P**hysical, **I**ntellectual, **E**motional health, maximizing individual **C**apabilities for quality of life, living **E**nvironment and **S**ocial including a person's beliefs, culture, and life story) offers an evidence‐based framework to address behavioural expressions through team collaboration. Building on our previous work addressing virtual application of the PIECES approach with Registered Practical Nurses (RPNs) leading as PIECES champions in LTC homes, this study aimed to co‐design a ToolKit (i.e., website, resources, infographics) with the collaboration of residents, their family/care partners, RPNs, administrators, PIECES mentors, and Behavioural Supports Ontario (BSO) Leads. The ToolKit will contain resources supporting virtual care planning including the PIECES approach.

**Method:**

This study employed a qualitative descriptive design. Four workshop sessions with two LTC homes in Ontario, Canada were held with residents, family/care partners, RPNs, and BSO Leads participating to co‐design a virtual ToolKit including the PIECES approach, and provide recommendations. Study collaborators attended sessions in person or by videoconferencing with the research team and PIECES mentors.

**Result:**

Recommendations for the information and web presence of the PIECES ToolKit included greater inclusion of infographics, embedded short video clips, reduced text, examples of communication memos and telephone scripts for engaging families, options to have the web page content read aloud, and practical user‐friendly tips to support communication technology (e.g., ZOOM) and preparation suggestions for virtual engagement for care planning. Further, separate sections specific to staff and family were advocated to meet unique needs, with limited available time, a range in familiarity with navigating websites, and improved to‐the‐point information.

**Conclusion:**

This project delivers an online accessible open resource available to guide staff and family in preparing for and implementing virtual team‐based care planning engaging family/care partners using the PIECES framework.

